# Burnout and Career Calling Profiles Among Nurses: Associations With Nursing Work Environment and Missed Nursing Care

**DOI:** 10.1155/jonm/1358852

**Published:** 2026-10-17

**Authors:** Nan Mo, Juan Ren, Dan Su, Feng Du, Xiyao Yang, Manzhen Bao, Miao Zhang, Qian Zhao, Zengzeng Wei

**Affiliations:** ^1^ Second Hospital of Anhui Medical University Hefei China; ^2^ School of Nursing Anhui Medical University Hefei China ahmu.edu.cn; ^3^ The Taikang Health and Wellness Industry Research Institute Anhui Medical University Hefei China ahmu.edu.cn; ^4^ Fuyang Cancer Hospital Fuyang China; ^5^ Nanjing Drum Tower Hospital Affiliated to Nanjing University Medical School Nanjing China

**Keywords:** burnout, career calling, latent profile analysis, missed nursing care, nurses, nursing work environment

## Abstract

**Background:**

Nurses are highly prone to burnout due to high pressure, heavy workloads, and emotional labor. Career calling has been associated with lower levels of burnout, but variable‐centered studies have overlooked heterogeneous subgroups among nurses.

**Objective:**

This person‐centered study used latent profile analysis to jointly examine career calling and burnout, identify burnout–career calling profiles, and explore factors associated with these profiles and their associations with self‐reported missed nursing care.

**Methods:**

A cross‐sectional study was conducted from March to September 2025 among 524 nurses. Participants completed the Maslach Burnout Inventory, Career Calling Scale, Nursing Work Environment Scale, and Missed Nursing Care Scale online. Latent profile analysis was applied.

**Results:**

Three profiles emerged: “Low Burnout–High Career Calling” (49.6%), “Reduced Personal Accomplishment” (37.8%), and “High Burnout–Low Career Calling” (12.6%). Age, educational level, professional title, and nursing work environment were associated with profile membership. Nurses in the “High Burnout–Low Career Calling” profile reported the highest level of missed nursing care, followed by those in the “Reduced Personal Accomplishment” profile, whereas those in the “Low Burnout–High Career Calling” profile showed the lowest level.

**Conclusion:**

This study identified three burnout–career calling profiles among nurses and examined their associated factors and differences in self‐reported missed nursing care.

**Implication for Nursing Management:**

Nursing managers could provide tailored support for the three burnout–career calling profiles at both organizational and individual levels, including creating a supportive work environment and offering psychological support. Future research could examine whether such tailored support may help reduce missed nursing care.

## 1. Introduction

Burnout is a consequence of chronic workplace stress and is characterized by emotional exhaustion, depersonalization, and reduced personal accomplishment [1]. Nurses are on the front line of patient care and are often required to make critical clinical decisions while providing both physical and psychological care to patients. Due to heavy workloads, stressful work environments, and emotional labor, nurses are particularly vulnerable to burnout [2, 3], with prevalence rates reportedly ranging from 11% to 56% [3]. Burnout can negatively affect nurses’ mental health, leading to depression and anxiety, and may also result in decreased job performance and increased turnover intention [1, 4]. Furthermore, burnout among nurses contributes to poorer quality of nursing care, compromises patient safety, and reduces patient satisfaction [3].

According to the Conservation of Resources (COR) theory, individuals’ initial resources facilitate the acquisition, protection, and maintenance of new resources, thereby promoting a positive gain spiral [5]. Individuals with greater psychological resources are better equipped to cope with risks and stressors [6]. Nurses may experience burnout because of the perceived or actual loss of four categories of resources: objects, conditions, personal characteristics, and energy [7]. Career calling refers to an intrinsic drive that enables nurses to find meaning in their work and motivates them to fulfill their professional responsibilities without expecting additional material rewards. As a positive psychological resource, career calling may alleviate burnout among nurses [8, 9]. Different levels of positive psychological resources (career calling) and negative psychological states (burnout) may coexist within individual nurses, reflecting distinct states of resource preservation or resource depletion, thereby leading nurses to exhibit different occupational psychological states. Traditional variable‐centered regression analysis cannot capture such heterogeneous combination patterns.

Latent profile analysis (LPA), a person‐centered analytical method, can identify distinct latent subgroups within a population. Previous person‐centered research has examined burnout profiles among nurses and their relationship with nursing work environment and care‐quality‐related outcomes [10]. However, previous studies have rarely examined the coexisting patterns of negative occupational psychological states and positive occupational psychological resources from a person‐centered perspective, nor have they sufficiently explored how these patterns are associated with nursing work environment or missed nursing care. In this study, we examined the interplay between negative occupational psychological states, represented by the three dimensions of burnout (emotional exhaustion, depersonalization, and reduced personal accomplishment), and a positive occupational psychological resource, namely, career calling. Different combinations of burnout and career calling levels may reflect distinct burnout–career calling patterns among nurses; therefore, LPA was used to jointly examine these two constructs and identify distinct burnout–career calling profiles.

According to the COR theory, personal characteristic resources do not develop in isolation; rather, their development and maintenance are highly dependent on the external environment [11]. The nursing work environment is defined as the organizational characteristics of the workplace that facilitate or constrain professional nursing practice, encompassing physical, social, and organizational factors [12]. A supportive nursing work environment, as a situational resource, can directly protect nurses’ emotional, cognitive, and attitudinal resources [13] and may be associated with higher levels of internal personal resources such as career calling, as well as with lower levels of burnout. Conversely, an unsupportive work environment is associated with higher levels of burnout [14]. Therefore, the nursing work environment, as a situational resource, may be associated with burnout–career calling profile membership.

Missed nursing care, first proposed by Kalisch et al. [15] in 2006, refers to any aspect of necessary patient care that is omitted or delayed [15, 16]. It is prevalent across various healthcare settings, including hospitals and community care settings [17]. Globally, the prevalence of missed nursing care among nurses ranges from 6.8% to 98.1% [16]. Missed nursing care threatens patient safety across countries and cultures and may lead to adverse events such as medication errors, infections, falls, and pressure ulcers. It can also delay patient recovery, prolong hospital stays, increase the risk of complications, and even significantly increase patient mortality [18]. Existing evidence suggests that burnout is closely associated with missed nursing care. Nurses experiencing burnout are reportedly five times more likely to report missed nursing care than those without burnout [19]. In contrast, career calling has been shown to reduce the occurrence of missed nursing care [8]. These findings suggest that nurses with different burnout–career calling profiles may exhibit significant differences in missed nursing care behaviors.

Based on the above findings, the nursing work environment, as a situational resource, may be associated with burnout–career calling profile membership; different profiles may reflect different states of resource preservation or resource depletion, and these different resource states may further be reflected in differences in missed nursing care. Therefore, this study used the total score of the Career Calling Scale and the three subscale scores of the Burnout Scale as joint indicators to identify latent profiles of burnout–career calling among clinical nurses. In addition, this study examined factors associated with latent profile membership, including nursing work environment and demographic variables, and compared differences in self‐reported missed nursing care across latent profiles. The findings of this study may provide evidence for developing targeted intervention strategies for nursing managers.

## 2. Materials and Methods

### 2.1. Study Design and Sample

This single‐center cross‐sectional study was conducted from March to September 2025 among clinical nurses recruited through convenience sampling from a tertiary hospital in Nanjing, China. The required sample size was determined based on the recommendation of Nylund‐Gibson and Choi [20], who suggested a minimum sample size of 300 for LPA. Considering an additional 20% allowance for invalid responses, the minimum required sample size was 375. Inclusion criteria were (a) registered nurses engaged in clinical practice and (b) at least 6 months of clinical nursing experience. Exclusion criteria were (a) nurses on rotation or internship in the study hospital; (b) nurses absent during the survey period due to external training or leave.

### 2.2. Instruments

#### 2.2.1. General Information Questionnaire

The questionnaire was designed by the researchers and included 10 items: age, gender, educational level, marital status, years of work experience, personal annual income, weekly overtime hours, number of night shifts per month, professional title, and employment form.

#### 2.2.2. Chinese Version of the Maslach Burnout Inventory

The Maslach Burnout Inventory‐Human Services Survey (MBI‐HSS), developed by Maslach and Jackson [21], was adapted into Chinese by Feng et al. [22]. It covers three dimensions: emotional exhaustion, depersonalization, and personal accomplishment, with a total of 22 items. A 7‐point Likert scale is used, with the frequency of occurrence ranging from “never” to “every day” scored from 0 to 6, respectively, giving a total score ranging from 0 to 132. Lower scores on the personal accomplishment dimension indicate a higher level of burnout, whereas higher scores on the emotional exhaustion and depersonalization dimensions indicate a higher level of burnout. To ensure that all dimensions were interpreted in the same direction, the personal accomplishment dimension was reverse‐scored and represented as reduced personal accomplishment in this study, with higher scores indicating a higher level of burnout. In this study, the overall Cronbach’s alpha coefficient of this scale was 0.817. Cronbach’s alpha coefficients for the three dimensions were 0.924 for emotional exhaustion, 0.798 for depersonalization, and 0.886 for reduced personal accomplishment.

#### 2.2.3. Calling Scale

The Calling Scale‐12 was developed by Dobrow et al. [23]. The scale consists of 12 items, rated on a 5‐point Likert scale ranging from 1 “strongly disagree” to 5 “strongly agree.” Total scores range from 12 to 60, with higher scores indicating a stronger sense of subjective value identification with one’s occupation and a higher level of intrinsic motivational drive toward work. The scale has demonstrated good reliability and validity. Mao et al. [24] first applied it to assess career calling among Chinese nurses, reporting a Cronbach’s alpha coefficient of 0.96. In this study, Cronbach’s alpha was 0.955.

#### 2.2.4. Chinese Version of the Nursing Work Environment Scale

This scale was developed by Shao et al. [25]. It consists of seven dimensions: professional autonomy, career development, nurse–physician relationships, leadership and management, basic security, adequate staffing, and recognition climate, with a total of 26 items. A 6‐point Likert scale is used, ranging from “strongly disagree” to “strongly agree,” scored from 1 to 6. The total score ranges from 26 to 156, with higher scores indicating a better nursing work environment. In this study, Cronbach’s alpha coefficient of the scale was 0.968. Cronbach’s alpha coefficients for the seven dimensions were 0.785 for professional autonomy, 0.875 for career development, 0.915 for nurse–physician relationships, 0.894 for leadership and management, 0.837 for basic security, 0.783 for adequate staffing, and 0.860 for recognition climate.

#### 2.2.5. Chinese Version of the Missed Nursing Care Scale

The scale was adapted into Chinese by Si et al. [26]. It consists of Part A (missed nursing care activities) and Part B (reasons for missed nursing care), which can be used independently. In this study, only Part A was used to measure missed nursing care, comprising 24 items. A 5‐point Likert scale is used, ranging from “never missed” to “always missed,” scored from 0 to 4. The total score ranges from 0 to 96, with higher scores indicating more frequent missed nursing care. In this study, Cronbach’s alpha coefficient for Part A was 0.968.

### 2.3. Data Collection

The purpose, significance, and design of the study were explained to the nursing administration department of one tertiary hospital in Nanjing. After obtaining institutional approval, an online survey link generated using Questionnaire Star was distributed to head nurses through WeChat groups. The head nurses subsequently forwarded the survey link to nurses’ WeChat groups. To minimize potential managerial pressure, selection bias, and social desirability bias associated with distribution by head nurses, standardized instructions were provided on the first page of the questionnaire, clearly stating that (1) participation was entirely voluntary; (2) the survey was anonymous, and nursing managers could not access any individual responses; and (3) data would be used solely for academic research purposes. In addition, the instructions detailed the study procedures, and participants completed the questionnaire after providing electronic informed consent. All questionnaire items were set as mandatory to ensure data completeness, and duplicate submissions from the same device or account were prevented. During the study period, a total of 2100 nurses were invited to participate. A total of 535 questionnaires were returned, yielding a response rate of 25.48%. Questionnaires with invalid response patterns or a completion time of less than 300 s were excluded. After independent verification by two researchers, 524 valid questionnaires were retained, yielding a valid questionnaire rate of 97.94%.

### 2.4. Ethical Considerations

This study was approved by the Ethics Committee of Nanjing Drum Tower Hospital, the Affiliated Hospital of Nanjing University Medical School (Approval No. 2025‐0111‐01). The study was conducted in accordance with the principles of voluntary participation and anonymity. All data were collected and used exclusively for academic research purposes.

### 2.5. Data Analysis

This study employed LPA to identify latent profiles of nurses’ burnout and career calling. LPA is a person‐centered approach used to identify unobserved heterogeneous subgroups within a population by assigning individuals to the latent profile for which they have the highest posterior probability of membership [27]. Analyses were conducted using Mplus version 8.3.

The Calling Scale‐12 used in this study is a unidimensional scale. Conceptually, this scale conceptualizes career calling as a holistic construct, and the total score can reflect an individual’s overall level of career calling. Therefore, the three dimensions of burnout and the total score of career calling were included as profile indicators in this study. For the burnout measure, higher scores on the emotional exhaustion and depersonalization dimensions indicate higher levels of burnout, whereas higher scores on the personal accomplishment dimension indicate lower levels of burnout. To ensure consistent interpretation across the three dimensions, the personal accomplishment subscale was reverse‐scored, and the dimension was relabeled as “reduced personal accomplishment”—that is, higher scores on this dimension reflect lower personal accomplishment and more severe burnout. Subsequently, all profile indicators (the three burnout dimensions and the total score of career calling) were standardized (z‐scored) to eliminate scaling effects and ensure that each indicator contributed equally to classification.

Models ranging from one‐to five‐profile solutions were estimated sequentially using maximum likelihood estimation with robust standard errors. Model fit was evaluated using the Akaike information criterion (AIC), Bayesian information criterion (BIC), and sample‐size‐adjusted Bayesian information criterion (aBIC). Lower AIC, BIC, and aBIC values indicate better model fit. The Lo–Mendell–Rubin likelihood ratio test (LMRT) and bootstrapped likelihood ratio test (BLRT) were used to compare models with different numbers of profiles. A significant *p*‐value (< 0.05) for the LMRT or BLRT indicates that the k‐profile model provides a significantly better fit than the (k − 1)‐profile model. Classification quality was comprehensively evaluated using entropy, average posterior probabilities (AvePP), and classification error rates (calculated as 1 − AvePP). Higher entropy and AvePP values (closer to 1) reflect better classification accuracy. Conventionally, entropy ≥ 0.80 and AvePP ≥ 0.70 were used as thresholds for adequate accuracy [28]. To avoid overextraction and ensure reliable parameter estimation, each profile was required to contain at least 5% of the total sample based on the most likely class membership [29, 30]. To reduce the likelihood of local maxima, 500 random sets of starting values were used in the initial stage, with the 100 best solutions retained for final optimization. The best loglikelihood value was replicated, indicating that the model converged to the global maximum solution. The final model was determined based on statistical fit indices, classification quality, profile sizes, substantive distinctiveness, theoretical consistency, and model parsimony.

After determining the optimal model, descriptive statistical analyses were conducted using SPSS version 24.0. None of the continuous variables were normally distributed; therefore, they were described using medians and interquartile ranges (IQRs). Categorical variables were described using frequencies and percentages.

The robust three‐step approach (R3STEP) in Mplus version 8.3 was used to examine predictors of latent profile membership, including demographic characteristics and the nursing work environment. All covariates were entered simultaneously into the model to estimate their unique effects on profile membership while controlling for other covariates. The BCH procedure in Mplus version 8.3 was used to examine differences in missed nursing care across latent profiles as a distal outcome. Pairwise comparisons were conducted to determine whether significant differences existed between profiles on the outcome variable. All statistical tests were two‐tailed, and statistical significance was set at *p* < 0.05.

## 3. Results

### 3.1. Descriptive Statistics

This study included 524 nurses. A total of three latent profiles were identified: 260 nurses (49.6%) were classified into the Low Burnout–High Career Calling profile, 66 (12.6%) into the High Burnout–Low Career Calling profile, and 198 (37.8%) into the reduced personal accomplishment profile. Table 1 presents the demographic characteristics of nurses across the three burnout–career calling profiles.

**TABLE 1:** Demographic characteristics of the profiles (*n* = 524).

Variable	Classification	Low Burnout–High Career Calling (*n* = 260, 49.6%)	High Burnout–Low Career Calling (*n* = 66, 12.6%)	Reduced Personal Accomplishment (*n* = 198, 37.8%)
		*n* (%)	*n* (%)	*n* (%)
Age (years)	< 35	128 (41.4)	45 (14.6)	136 (44.0)
	35 –< 45	90 (58.4)	14 (9.1)	50 (32.5)
	≥ 45	42 (68.9)	7 (11.5)	12 (19.7)

Gender	Male	7 (43.8)	2 (12.5)	7 (43.8)
	Female	253 (49.8)	64 (12.6)	191 (37.6)

Education level	Bachelor’s degree or below	257 (50.7)	60 (11.8)	190 (37.5)
	Master’s degree or above	3 (17.6)	6 (35.3)	8 (47.1)

Marital status	Married	212 (52.2)	51 (12.6)	143 (35.2)
	Single	48 (40.7)	15 (12.7)	55 (46.6)

Years of work experience	< 5	30 (33.7)	16 (18.0)	43 (48.3)
	5 –< 10	49 (42.6)	14 (12.2)	52 (45.2)
	≥ 10	181 (56.6)	36 (11.3)	103 (32.2)

Annual income (10,000 CNY)	< 5	44 (57.1)	11 (14.3)	22 (28.6)
	5 –< 10	101 (45.5)	29 (13.1)	92 (41.4)
	≥ 10	115 (51.1)	26 (11.6)	84 (37.3)

Weekly overtime hours	0 –< 5	193 (51.3)	48 (12.8)	135 (35.9)
	≥ 5	67 (45.3)	18 (12.2)	63 (42.6)

Number of night shifts per month	0–4	135 (60.8)	29 (13.1)	58 (26.1)
	5–8	68 (42.0)	19 (11.7)	75 (46.3)
	≥ 9	57 (40.7)	18 (12.9)	65 (46.4)

Professional Title	Nurse	10 (50.0)	5 (25.0)	5 (25.0)
	Senior Nurse	76 (43.2)	22 (12.5)	78 (44.3)
	Nursing Supervisor	119 (47.8)	34 (13.7)	96 (38.6)
	Associate chief nurse or above	55 (69.6)	5 (6.3)	19 (24.1)

Employment status	Nonestablished	94 (43.3)	32 (14.7)	91 (41.9)
	Established	166 (54.1)	34 (11.1)	107 (34.9)

Nursing Work Environment	Total score	131.00 (126.00, 148.75)	109.00 (102.00, 123.00)	118.00 (106.00, 128.00)

### 3.2. LPA of Burnout–Career Calling Among Nurses

First, LPA models with one to five classes were estimated based on the three dimensions of burnout and the total career calling score. When the number of profiles increased from three to four, the AIC, BIC, and aBIC continued to decrease, and the BLRT remained statistically significant (*p* < 0.001). However, the LMRT was not significant (*p* > 0.05), indicating that the four‐profile solution did not significantly improve model fit compared with the three‐profile solution. The AvePP values for the four‐ and three‐profile solutions were both above 0.70, and the entropy values were both above 0.80 (0.858 for the four‐profile solution and 0.824 for the three‐profile solution), indicating acceptable classification quality for both solutions. When the model was extended to five profiles, the AIC, BIC, and aBIC reached their lowest values, and both the BLRT and LMRT were statistically significant (*p* < 0.001 and *p* < 0.05, respectively), with the smallest profile accounting for 5.9% of the sample, exceeding the 5% threshold. The five‐profile solution had an entropy of 0.853 and AvePP values of 0.847–0.947, also indicating acceptable classification quality. LPA model fit indices and class probabilities are shown in Table 2. Classification quality indices for the three‐, four‐, and five**-**profile solutions are presented in Table 3.

**TABLE 2:** LPA model fit indices and class probabilities.

Class	AIC	BIC	ABIC	Entropy	*p*‐value		Category probability (%)
					LMRT	BLRT	
1	5960.187	5994.278	5968.884	—	—	—	—
2	5448.793	5504.192	5462.927	0.801	< 0.001	< 0.001	63.4/36.6
3	5194.468	5271.175	5214.039	0.824	< 0.001	< 0.001	37.8/49.6/12.6
4	5061.696	5159.710	5086.703	0.858	0.2497	< 0.001	9.5/33.0/46.8/10.7
5	4966.827	5086.149	4997.270	0.853	0.0123	< 0.001	43.9/23.9/10.3/5.9/16.0

Abbreviations: ABIC, adjusted Bayesian information criterion; AIC, Akaike information criterion; BIC, Bayesian Information Criterion; BLRT, Bootstrapped likelihood ratio test; LMRT, Lo–Mendell–Rubin likelihood ratio test.

**TABLE 3:** Classification quality indices for the three‐, four‐, and five‐profile solutions.

Class	AvePP	Classification error rate (%)
3	0.900–0.937	6.3–10.0
4	0.873–0.962	3.8–12.7
5	0.847–0.947	5.3–15.3

Abbreviation: AvePP, average posterior probabilities.

To further evaluate whether the additional profiles in the four‐ and five‐profile solutions exhibited substantive distinctiveness, we compared the standardized mean patterns of the profiles in the three‐, four‐, and five‐profile solutions (see Figures 1–3). Based on the characteristic patterns of the three profiles, they were named the “Low Burnout–High Career Calling” profile, the “Reduced Personal Accomplishment” profile, and the “High Burnout–Low Career Calling” profile, respectively.

**FIGURE 1:**
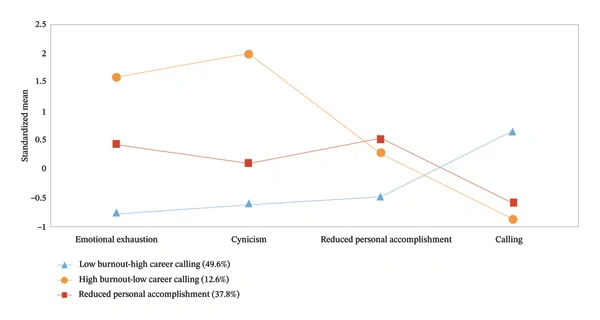
Three latent profiles among nurses.

**FIGURE 2:**
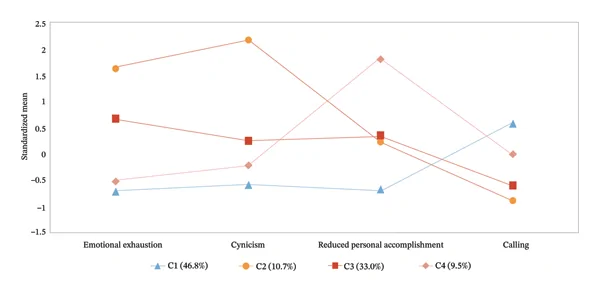
Four latent profiles among nurses.

**FIGURE 3:**
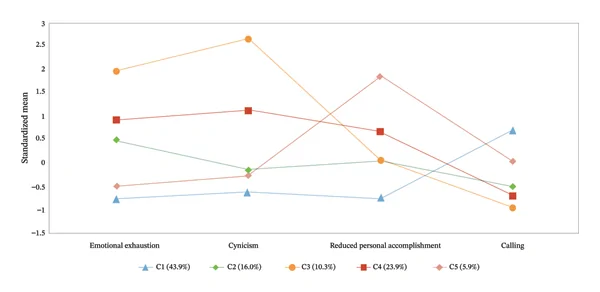
Five latent profiles among nurses.

In the four‐profile solution, the newly added C4 was mainly characterized by a relatively high level of reduced personal accomplishment, and its overall pattern was fairly similar to that of the “Reduced Personal Accomplishment” profile in the three‐profile solution. The two profiles showed similar patterns on the reduced personal accomplishment dimension; the main difference was that C4 exhibited higher levels of both reduced personal accomplishment and career calling, and relatively lower levels of emotional exhaustion and depersonalization. Therefore, C4 was more likely to reflect a further subdivision of the “Reduced Personal Accomplishment” pattern within the same dimension rather than constituting a new profile with a clearly distinct overall pattern.

In the five‐profile solution, C1 and C3 were highly consistent with the “Low Burnout–High Career Calling” and “High Burnout–Low Career Calling” profiles in the three‐profile solution, respectively. C2, C4, and C5 did not constitute clearly distinct profiles beyond those identified in the three‐profile solution. Specifically, C2 and C4 combined features of the two‐core profiles, “High Burnout–Low Career Calling” and “Reduced Personal Accomplishment”; C5 was mainly characterized by a relatively high level of reduced personal accomplishment and was therefore most similar to the “Reduced Personal Accomplishment” profile. Therefore, although C2, C4, and C5 differed in the specific levels of various indicators, they did not form new profiles that were theoretically meaningful and substantively distinct from the existing profiles.

The three profiles could be interpreted within the COR theoretical framework. Specifically, nurses in the “Low Burnout–High Career Calling” profile exhibited low levels of emotional exhaustion and depersonalization, along with high levels of personal accomplishment and career calling. Nurses in the “Reduced Personal Accomplishment” profile exhibited moderate levels of emotional exhaustion, depersonalization, and career calling but relatively low levels of personal accomplishment. Nurses in the “High Burnout–Low Career Calling” profile exhibited high levels of emotional exhaustion and depersonalization, moderate levels of personal accomplishment, and low levels of career calling. Within the COR framework, these patterns may be interpreted as different combinations of positive psychological resources and negative psychological states at a single time point. However, because resources were not measured longitudinally, these interpretations remain hypothetical and should be tested in future longitudinal research.

Although the five‐profile solution showed better statistical fit, the additional profiles did not demonstrate sufficient substantive distinctiveness or theoretical interpretability. Accordingly, after comprehensively considering substantive distinctness, theoretical consistency, model parsimony, statistical fit, classification quality, and profile size, this study ultimately retained the three‐profile solution. Although the three‐profile solution did not have the lowest information criterion values, it provided a relatively parsimonious representation of three clearly and substantively distinct profiles.

### 3.3. Factors Associated With Latent Profile Membership Based on the R3STEP Analysis

Because conceptual overlap among age, years of work experience, and professional title was a potential concern, collinearity diagnostics were conducted before model estimation. Collinearity diagnostics were therefore performed prior to model estimation. The variance inflation factor (VIF) values ranged from 1.443 to 2.500, all below the threshold of 5, indicating no serious multicollinearity. Nevertheless, to simplify the model and avoid conceptual redundancy, age and professional title were retained, whereas years of work experience was excluded, as the former two reflect nurses’ demographic characteristics and professional hierarchy, respectively, offering greater representativeness and distinctiveness. This decision was based on conceptual considerations rather than variable selection based on statistical significance or VIF results. Subsequently, a multinomial logistic regression analysis using the R3STEP approach was performed to examine the associations between covariates and latent profile membership, with the High Burnout–Low Career Calling profile as the reference category. Compared with the high burnout–low career calling profile, nurses aged 35 –< 45 years were more likely to be classified into the Low Burnout–High Career Calling profile (*B* = 1.106, SE = 0.515, OR = 3.022, 95% CI: 1.101–8.297). Compared with nurses with a bachelor’s degree or below, those with a master’s degree or above had lower odds of being classified into the Low Burnout–High Career Calling profile compared with the High Burnout–Low Career Calling profile (*B* = −2.483, SE = 0.910, OR = 0.084, 95% CI: 0.014–0.498). In addition, nurses with a professional title of associate chief nurse or above were more likely to belong to the reduced personal accomplishment profile (*B* = 2.645, SE = 1.310, OR = 14.084, 95% CI: 1.082–183.370). Importantly, higher C‐NWES scores were significantly associated with increased odds of being classified into both the Low Burnout–High Career Calling profile (OR = 1.151, 95% CI: 1.105–1.199) and the reduced personal accomplishment profile (OR = 1.026, 95% CI: 1.000–1.052), compared with the High Burnout–Low Career Calling profile (see Table 4).

**TABLE 4:** Results of the R3STEP analysis of factors associated with latent profile membership.

Variables	Low Burnout–High Career Calling vs High Burnout–Low Career Calling			Reduced Personal Accomplishment vs High Burnout–Low Career Calling		
	B (SE)	*p*	OR (95% CI)	B (SE)	*p*	OR (95% CI)
*Age (years) (Ref. < 35)*						
35 ‐< 45	1.106 (0.515)	**0.032**	3.022 [1.101, 8.297]	0.216 (0.487)	0.657	1.241 [0.477, 3.227]
≥ 45	−0.256 (0.874)	0.770	0.774 [0.140, 4.293]	−1.232 (0.919)	0.180	0.292 [0.048, 1.765]

*Gender (Ref. Female)*						
Male	0.118 (1.303)	0.928	1.125 [0.087, 14.500]	−0.181 (1.250)	0.885	0.835 [0.072, 9.687]

*Education level (Ref. Bachelor’s degree or below)*						
Master’s degree or above	−2.483 (0.910)	**0.006**	0.084 [0.014, 0.498]	−0.191 (0.731)	0.794	0.827 [0.197, 3.460]

*Marital status (Ref. Married)*						
Single	−0.852 (0.626)	0.173	0.426 [0.125, 1.455]	0.302 (0.458)	0.510	1.352 [0.551, 3.317]

*Annual income (10,000 CNY) (Ref. < 5)*						
5 –< 10	−0.502 (0.569)	0.377	0.605 [0.198, 1.847]	0.299 (0.535)	0.576	1.349 [0.472, 3.852]
≥ 10	−0.320 (0.601)	0.595	0.726 [0.224, 2.356]	0.329 (0.571)	0.564	1.390 [0.454, 4.251]

*Weekly overtime hours (Ref. 0 –< 5)*						
≥ 5	−0.699 (0.473)	0.139	0.497 [0.197, 1.256]	0.187 (0.423)	0.659	1.205 [0.526, 2.761]

*Number of night shifts per month (Ref. 0–4)*						
5–8	0.190 (0.524)	0.718	1.209 [0.433, 3.378]	0.854 (0.487)	0.079	2.349 [0.904, 6.105]
≥ 9	0.136 (0.533)	0.799	1.145 [0.403, 3.252]	0.784 (0.502)	0.118	2.189 [0.818, 5.857]

*Professional Title (Ref. Nurse)*						
Senior Nurse	0.945 (1.369)	0.490	2.574 [0.176, 37.740]	1.421 (0.861)	0.099	4.141 [0.765, 22.400]
Nursing Supervisor	0.457 (1.452)	0.753	1.579 [0.092, 27.040]	1.332 (0.935)	0.154	3.789 [0.606, 23.690]
Associate chief nurse or above	2.425 (1.692)	0.152	11.306 [0.406, 314.600]	2.645 (1.310)	**0.044**	14.084 [1.082, 183.370]

*Employment status (Ref. Non-established)*						
Established	−0.287 (0.505)	0.571	0.751 [0.279, 2.021]	0.107 (0.462)	0.817	1.113 [0.450, 2.751]
C‐NWES	0.141 (0.021)	**< 0.001**	1.151 [1.105, 1.199]	0.025 (0.013)	**0.044**	1.026 [1.000, 1.052]

*Note:* C‐NWES: Chinese Version of the Nursing Work Environment Scale; C‐NWES was entered as a continuous total score (range, 26–156). The ORs shown in the table represent a 1‐point increase in the C‐NWES score. For ease of interpretation, ORs and 95% CIs for larger increments were calculated from the original regression coefficients and standard errors. A 5‐point increase corresponds to ORs of 2.024 (95% CI, 1.647–2.486) for Low Burnout–High Career Calling versus High Burnout–Low Career Calling and 1.133 (95% CI, 0.998–1.287) for reduced personal accomplishment versus high burnout–low career calling. The bold values indicate statistically significant differences, representing *p* < 0.05 or *p* < 0.001.

### 3.4. Differences in Self‐Reported Missed Nursing Care Across Latent Profiles

The BCH analysis showed significant differences in self‐reported missed nursing care across the identified latent profiles (BCH *χ* ^2^ = 73.753, *p* < 0.001). Nurses in the High Burnout–Low Career Calling profile reported the highest level of missed nursing care (mean = 20.159, SE = 2.340, 95% CI: 15.574–24.744), followed by the reduced personal accomplishment profile (mean = 13.931, SE = 0.927, 95% CI: 12.114–15.748), whereas the Low Burnout–High Career Calling profile reported the lowest level (mean = 6.122, SE = 0.590, 95% CI: 4.966–7.278).

Pairwise comparisons indicated that the High Burnout–Low Career Calling profile had significantly higher self‐reported missed nursing care scores than both the Low Burnout–High Career Calling profile (*χ* ^2^ = 34.001, *p* < 0.001) and the reduced personal accomplishment profile (*χ* ^2^ = 5.701, *p* < 0.05). In addition, the reduced personal accomplishment profile also reported significantly higher missed nursing care than the Low Burnout–High Career Calling profile (*χ* ^2^ = 44.445, *p* < 0.001) (see Table 5). Pairwise comparisons were conducted using BCH‐adjusted Wald tests. No multiple comparison correction was applied, given the limited number of pairwise comparisons (three comparisons); therefore, the findings were interpreted cautiously. Figure 4 illustrates the self‐reported missed nursing care scores across the different latent profiles.

**TABLE 5:** Differences in self‐reported missed nursing care across latent profiles.

Profile	Mean (SE)	95% CI	BCH *χ* ^2^	Comparison of different profiles
High Burnout–Low Career Calling	20.159 (2.340)	15.574–24.744	73.753^∗∗^	High Burnout–Low Career Calling > Reduced Personal Accomplishment > Low Burnout–High Career Calling
Low Burnout–High Career Calling	6.122 (0.590)	4.966–7.278	—	
Reduced Personal Accomplishment	13.931 (0.927)	12.114–15.748	—	

High Burnout–Low Career Calling vs. Low Burnout–High Career Calling	—	—	34.001^∗∗^	—

High Burnout–Low Career Calling vs. Reduced Personal Accomplishment	—	—	5.701^∗^	—

Low Burnout–High Career Calling vs. Reduced Personal Accomplishment	—	—	44.445^∗∗^	—

^∗^ *p*  <  0.05.

^∗∗^ *p*  <  0.001.

**FIGURE 4:**
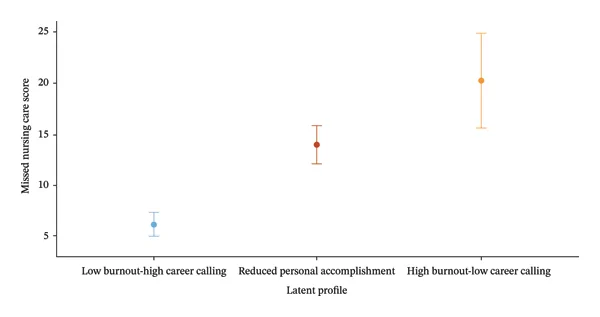
Comparison of self‐reported missed nursing care scores among latent profiles.

## 4. Discussion

This study identified three latent profiles of burnout–career calling among nurses, namely, the “Low Burnout–High Career Calling,” “Reduced Personal Accomplishment,” and “High Burnout–Low Career Calling” profiles. Age, education level, professional title, and nursing work environment were found to be associated with profile membership. Furthermore, significant differences in self‐reported missed nursing care scores were found across nurses with different profiles.

### 4.1. Latent Profiles of Burnout–Career Calling Among Nurses

This study identified three profiles of burnout–career calling among nurses, providing a person‐centered understanding of their burnout–career calling patterns. According to the COR theory, individuals possess limited resources and will make every effort to acquire, preserve, and maintain these resources. Any perceived or actual loss of resources can cause psychological stress [5]. Faced with high workloads and high‐pressure environments in clinical practice, nurses often need to expend excessive physical and emotional energy, resulting in a state of prolonged resource depletion. Over time, nurses become physically and emotionally exhausted, which may evolve into burnout. Career calling is defined as a strong passion for one’s work and identification with its goals, representing a positive psychological resource [23]. This study combined burnout and career calling in LPA, revealing person‐centered burnout–career calling profiles reflecting different combinations of indicators of negative occupational psychological states and positive occupational psychological resources among nurses. Because this study used a cross‐sectional design, it could not determine whether individuals’ profile membership remained stable over time or whether nurses transitioned between profiles. Therefore, future research could use longitudinal latent transition analysis to further examine the dynamic transitions among profiles.

Nurses of the “Low Burnout–High Career Calling” profile exhibited lower levels of emotional exhaustion and depersonalization, as well as higher levels of personal accomplishment and career calling. This suggests that these nurses experience less depletion of emotional resources, possess a strong passion for their work, and are able to derive a sense of achievement from it. These nurses may benefit from a supportive work environment and demonstrate high levels of psychological resilience [1].

Nurses in the “Reduced Personal Accomplishment” profile exhibited moderate levels of emotional exhaustion and depersonalization, a low level of personal accomplishment, and a moderate level of career calling. This profile suggests that although these nurses maintained a moderate level of career calling and experienced moderate levels of emotional exhaustion and depersonalization, they reported a relatively low sense of personal accomplishment. Compared with the “High Burnout–Low Career Calling” profile, their levels of emotional exhaustion and depersonalization were not as high, and their level of career calling was not the lowest; however, their sense of personal accomplishment remained relatively low. Therefore, interventions for nurses in this profile should not only address emotional exhaustion and depersonalization but also place greater emphasis on recognizing their professional competence, reinforcing their sense of professional value, and strengthening their sense of personal accomplishment, thereby helping them maintain positive occupational resources and supporting more adaptive occupational functioning.

Nurses in the “High Burnout–Low Career Calling” profile exhibited high levels of emotional exhaustion and depersonalization, a moderate level of personal accomplishment, and a low level of career calling. Notably, their level of personal accomplishment was not the lowest among the identified profiles, which may reflect the fact that the three dimensions of burnout and career calling do not necessarily vary in parallel and may be influenced by different factors. This finding is consistent with previous research [1]. Even when nurses experience high levels of emotional exhaustion and depersonalization and low levels of career calling, their existing professional competence, work experience, or professional identity may still help maintain a moderate level of personal accomplishment to some extent. Previous research has shown that nurses’ burnout may be related to systemic stressors, including promotion pressure, clinical workload, organizational demands, and work–family conflict [31]. Combined with the profile patterns observed in this study and previous research, interventions for nurses in the “High Burnout–Low Career Calling” profile could consider alleviating their emotional exhaustion and depersonalization by creating a supportive work environment and providing psychological support. In addition, enhancing career calling may be an intervention target worthy of testing in future research. Interventions could emphasize the value and positive impact of nursing work and help nurses recognize the meaning embedded in their professional roles, thereby strengthening their career calling and sense of personal accomplishment [32, 33].

### 4.2. Factors Associated With Latent Profile Membership

The results showed that, compared with the “High Burnout–Low Career Calling” profile, nurses aged 35–< 45 years were more likely to be classified as the “Low Burnout–High Career Calling” profile. In addition, compared with the “Low Burnout–High Career Calling” profile, nurses with a master’s degree or higher were more likely to be classified into the “High Burnout–Low Career Calling” profile. Furthermore, nurses with a professional title of associate chief nurse or above were more likely to be classified as the “Reduced Personal Accomplishment” profile. Most importantly, compared with the “High Burnout–Low Career Calling” profile, nurses working in a better nursing work environment were more likely to be classified as the “Low Burnout–High Career Calling” and “Reduced Personal Accomplishment” profiles.

Nurses aged 35 –< 45 years were more likely to be classified as the “Low Burnout–High Career Calling” profile. This finding is consistent with previous studies [34, 35]. Compared with younger nurses, those aged 35 –< 45 years have accumulated many years of work experience, possess strong professional skills and abilities, and have developed a relatively stable professional identity. In addition, they often have established mature collegial relationships and family support, enabling them to effectively balance work and family, buffer occupational stress, and thus demonstrate higher adaptability [36]. On the other hand, nurses who have been in a prolonged state of burnout may have already left the profession [37]; therefore, among nurses still working in clinical settings within this age group, the proportion of those with burnout may be relatively smaller.

Compared with the “Low Burnout–High Career Calling” profile, nurses with a master’s degree or above were more likely to be classified into the “High Burnout–Low Career Calling” profile. This finding is consistent with the results reported by Li et al. [36]. One possible explanation is that nurses with higher levels of education may have greater career aspirations and higher professional standards and thus may face greater work pressure, thereby increasing their risk of burnout [36, 38]. However, this finding should be interpreted with caution because the subgroup with a master’s degree or above was relatively small, which may lead to imprecise estimates; therefore, this potential explanation needs to be confirmed in a larger sample.

Compared with the “High Burnout–Low Career Calling” profile, nurses with the professional title of associate chief nurse or above were more likely to be classified into the “Reduced Personal Accomplishment” profile. This finding is consistent with previous research [1]. Although a relatively large proportion of nurses with higher professional titles were classified into the “Low Burnout–High Career Calling” profile, this profile itself accounted for the largest proportion of the total sample, and no statistically significant association was observed between higher professional title and membership in the “Low Burnout–High Career Calling” profile; therefore, this descriptive pattern should not be overinterpreted. Nurses with higher professional titles appeared more likely to belong to the “Reduced Personal Accomplishment” profile rather than the “High Burnout–Low Career Calling” profile. One possible explanation is that this may be related to the greater role responsibilities and higher work demands that this group may face [1, 39, 40]. However, the above mechanisms were not measured in this study, and some professional title subgroups had relatively small sample sizes and wide confidence intervals; therefore, further investigation in future research is still needed.

Compared with the “High Burnout–Low Career Calling” profile, nurses working in a better nursing work environment were more likely to be classified as the “Low Burnout–High Career Calling” and “Reduced Personal Accomplishment” profiles. This finding is consistent with previous studies [34, 41, 42]. A favorable nursing work environment, as a contextual resource encompassing adequate staffing, nurse participation in decision‐making, positive relationships with physicians and managers, recognition, and sufficient resources and support [34, 41, 42], may be related to nurses’ high levels of personal psychological resources (such as career calling) and lower levels of emotional exhaustion and depersonalization. However, the organizational and contextual resources provided by a favorable work environment do not necessarily correspond directly to a high level of personal accomplishment. Some nurses may avoid experiencing pronounced burnout because of a more supportive work environment, yet continue to experience a reduced sense of personal accomplishment due to limited career development opportunities, insufficient perceived value in their work, or a mismatch between their capabilities and career expectations, thereby being more likely to be classified into the “Reduced Personal Accomplishment” profile. This finding suggests that, in addition to improving the nursing work environment through measures such as flexible scheduling, adequate staffing, workload reduction, and encouraging nurses to participate in decision‐making, nursing managers could also pay greater attention to nurses’ career development, recognition of their professional competence, and enhancement of their sense of accomplishment.

### 4.3. Differences in Nurses’ Self‐Reported Missed Nursing Care Across Profiles

The results showed that nurses in the “High Burnout–Low Career Calling” profile reported the highest level of missed nursing care, followed by those in the “Reduced Personal Accomplishment” profile, while those in the “Low Burnout–High Career Calling” profile reported the lowest level. This finding is consistent with previous studies [8, 19]. In this study, nurses in the “Reduced Personal Accomplishment” profile were characterized only by low personal accomplishment, with the other dimensions at moderate levels. Some scholars have suggested that the personal accomplishment dimension of burnout differs from the other two dimensions, and that emotional exhaustion and depersonalization may have a stronger influence on nursing outcomes, such as patient safety, than personal accomplishment [3]. This may help explain why the level of self‐reported missed nursing care in this profile was intermediate between those in the “Low Burnout–High Career Calling” and “High Burnout–Low Career Calling” profiles, being significantly higher than that in the former but lower than that in the latter. The “Low Burnout–High Career Calling” profile was associated with the lowest level of self‐reported missed nursing care, suggesting that effective measures to alleviate emotional exhaustion and depersonalization, preserve nurses’ personal accomplishment, and strengthen career calling may help reduce nurses’ self‐reported missed nursing care. Future research using longitudinal or intervention designs could further examine the effectiveness of interventions targeting the nursing work environment, burnout, and career calling.

## 5. Conclusion

Using a person‐centered approach that jointly considered career calling and burnout, this study identified three latent profiles of burnout–career calling among nurses: “Low Burnout–High Career Calling,” “Reduced Personal Accomplishment,” and “High Burnout–Low Career Calling.” Age, educational level, professional title, and nursing work environment were associated with profile membership, and these profiles differed in levels of self‐reported missed nursing care.

### 5.1. Implications for Nursing Management

This study identified three profiles of burnout–career calling among nurses, suggesting that nursing management could consider the characteristics of different profiles and provide targeted support at both individual and organizational levels.

At the organizational level, for nurses in the “High Burnout–Low Career Calling” profile, strategies could include reducing job demands by optimizing work schedules, ensuring adequate staffing, and reducing workload while creating a respectful and supportive work environment. For nurses in the “Reduced Personal Accomplishment” profile, it may be helpful to provide clear career development pathways, regular performance feedback, and recognition of professional competence, which may help enhance their personal accomplishment. For nurses in the “Low Burnout–High Career Calling” profile, efforts could focus on providing ongoing career development opportunities.

At the individual level, for nurses in the “High Burnout–Low Career Calling” profile, nursing managers could pay greater attention to their emotional health and provide personalized psychological support and stress management training, helping them recognize early signs of burnout and develop effective coping strategies. For nurses in the “Reduced Personal Accomplishment” profile, psychological support could be provided to help them gradually rebuild career confidence and personal accomplishment. Nurses in the “Low Burnout–High Career Calling” profile could be encouraged to sustain their career enthusiasm.

This study also observed that these profiles differed in levels of self‐reported missed nursing care. Future research could further explore whether support strategies tailored to different profiles may help reduce missed nursing care.

### 5.2. Limitations

This study has several limitations. First, self‐report questionnaires from a single online survey were used, which may introduce social desirability bias, recall bias, and common method bias. Future research should consider incorporating independent organizational indicators, staffing measures, administrative data, or objectively recorded missed nursing care to verify these findings and further clarify the relationships among variables. Second, the participation rate of nurses in this study was only 25.48%, indicating a substantial possibility of nonresponse bias. Because basic characteristics of invited nurses who did not respond were not collected, we were unable to compare respondents with the source nursing workforce, which limits our ability to assess nonresponse bias. The estimated profile prevalence may not represent the nursing workforce in the invited hospitals, and these proportions should be interpreted with caution. Third, the cross‐sectional design precludes causal inferences. Longitudinal studies are needed to better understand the dynamic relationships among burnout, career calling, and missed nursing care. Fourth, given the single‐hospital convenience sample used in this study, the latent profiles identified may vary across regions, hospital levels, specialties, and healthcare systems, which may limit the external validity and generalizability of the findings. Future studies should consider expanding the sample to improve generalizability.

## Author Contributions

Zengzeng Wei: conceptualization, methodology, and project administration.

Qian Zhao: conceptualization, methodology, and project administration.

Nan Mo: conceptualization, methodology, and writing–original draft.

Juan Ren: conceptualization and writing–review and editing.

Dan Su: conceptualization and writing–review and editing.

Feng Du: writing–review and editing.

Xiyao Yang: writing–review and editing.

Manzhen Bao: writing–review and editing.

Miao Zhang: writing–review and editing.

Nan Mo, Juan Ren, and Dan Su are co‐first authors.

## Funding

This study was supported by Nursing Project of Anhui Institite of Translational Medicine, 2025zhyx‐hl‐A19, 2024zhyx‐hl‐A04.

## Disclosure

The funders had no role in the study design, data collection, analysis, interpretation, writing, or the decision to submit the manuscript for publication.

## Conflicts of Interest

The authors declare no conflicts of interest.

## Data Availability

The data that support the findings of this study are available from the corresponding author upon reasonable request. The data are not publicly available due to privacy or ethical restrictions.
